# Perspectives of working-age adults with aphasia regarding social participation

**DOI:** 10.4102/ajod.v9i0.713

**Published:** 2020-12-15

**Authors:** Nadia M. Souchon, Esedra Krüger, Renata Eccles, Bhavani S. Pillay

**Affiliations:** 1 Department of Speech-Language Pathology and Audiology, Faculty of Humanities, University of Pretoria, Pretoria, South Africa

**Keywords:** social participation, aphasia, lower and middle-income countries, stroke, working-age adults

## Abstract

**Background:**

Working-age adults with aphasia experience difficulties in social participation, specifically fulfilling social roles and reintegrating into communities. Literature regarding social participation of people with aphasia (PWA) is predominantly based on studies conducted in high-income countries (HIC), limiting generalisability of findings. Perspectives of social participation are influenced by person, place and cultural background warranting investigation in heterogeneous low- and middle-income countries (LMICs), like South Africa.

**Objectives:**

Describe perspectives of working-age adults with aphasia regarding social participation within the first 2 years post-incident.

**Method:**

Semi-structured interviews gained perspectives of 10 working-age adults (with mild to moderate aphasia) using principles of supported conversation for adults with aphasia. Data were thematically analysed to describe participants’ perspectives of social participation.

**Results:**

Seven themes were identified pertaining to participants’ perspectives of social participation. Participants considered rehabilitation services, faith-related activities and returning to work as valued areas of social participation. Previous interests, presence of support and characteristics of communication partners determined their preference and willingness to participate with others. Finally, personal attitudes and feelings continued to influence their perspectives of social participation, as well as their motivation to participate.

**Conclusion:**

Successful social participation was dependent on the PWA’s perceived value of social activities and presence of support from significant others. Speech-language therapists are in the ideal position to facilitate PWA’s communication abilities and their experience of successful participation through the implementation of person-centered care and community-led intervention. This study provided a preliminary investigation of social participation in South Africa and further investigation is warranted.

## Introduction

People with aphasia (PWA) often experience difficulty fully participating in social situations and everyday activities because of their acquired language impairments (Alary Gauvreau & Le Dorze 2020). As language is the medium in which human interactions occur, symptoms of aphasia have far-reaching consequences than mere communication difficulties, indicating the crucial role communication abilities play in social participation (Woelders et al. 2018). People with aphasia experience decreased exposure to social situations as well as challenges when attempting to participate in meaningful daily tasks, often leading to social isolation (Fotiadou et al. 2014). Additionally, communication difficulties may result in changes in their relationships with family and community members contributing to a decline in social activities (Kusambiza-Kiingi, Maleka & Ntsiea 2017).

People with aphasia’s social experiences are largely defined by context, specifically cultural backgrounds and beliefs. This influences the manner in which PWA may react to their diagnosis as well as their community’s attitudes towards disability affecting social participation (Penn & Armstrong 2017). Community organisation and personal attitudes differ according to person, place and cultural background, warranting the adoption of an anthropological and holistic perspective when investigating an individual’s experience of disability and their ability to reintegrate into everyday life (Legg & Penn 2014). Clinical practice and service-delivery frameworks are often developed in the high-income countries (HIC) that have access to increased resources and services (Gonzalez-Suarez et al. 2012). Health care professionals in low- and middle-income countries (LMIC) encounter challenges during service-delivery as frameworks may not be contextually relevant because of differing health care systems, limited resources and a contrast in patient’s overall needs (Gonzalez-Suarez et al. 2012). These challenges may be heightened in South Africa where there is a multicultural society, quadruple burden of disease and limited context-specific resources (Bradshaw et al. 2019; Gonzalez-Suarez et al. 2012). Continued research on aphasia rehabilitation in South Africa is warranted to develop and provide holistic context-specific frameworks to guide healthcare professionals during intervention (Masuku, Mophosho & Tshabalala 2018). Therefore, healthcare professionals must consider PWA’s perspectives of meaningful engagement and willingness to participate, alongside their cultural context, community attitudes and beliefs and accessibility to their environment (Laliberté, Alary Gauvreau & Le Dorze 2016; Penn & Armstrong 2017).

Social participation is regarded as one of the most important determinants of successful rehabilitation, yet its definition is still unclear (De Souza et al. 2017). The World Health Organisation’s (WHO) International Classification of Functioning, Disability and Health Framework [ICF] (World Health Organization [WHO] 2001) encourages speech-language therapists (SLTs) and other healthcare professionals to acknowledge the multitude of factors involved in PWA’s rehabilitation process. Social participation is largely defined by an individual’s successful involvement in social activities including engagement in personal relationships and community life, specific to the context in which they live (Laliberté et al. 2016). Successful social participation is difficult to achieve for PWA because of their communication difficulties, as social participation is reliant on effective communication between individuals and significant others. This leads to difficulties with interpersonal relationships, accessibility to community life and employment for PWA (Alary Gauvreau & Le Dorze 2020).

Social participation comprises of dynamic and complex individual factors that are influenced by personal, environmental and social factors (Woodman et al. 2014). Research conducted within the South African context found an indisputable relationship between an individual’s experience of aphasia with their social and cultural values and beliefs (Legg & Penn 2012). South Africa, with its culturally and linguistically diverse population, may have reports of differing experiences of aphasia and contrasting perspectives of social participation, when compared to HIC (Watermeyer 2019). The importance of acknowledging culture and context when attempting to understand social participation for PWA is recognised in literature, although the majority of research is from HIC (Pike, Kritzinger & Pillay 2017). There is a need for further investigation from heterogeneous LMIC, such as South Africa. The profile and experiences of PWA in LMIC may differ to those in HIC, as contextual experiences are embedded in individuals’ understanding of illness; influencing PWA’s response to aphasia, attitudes towards the future and the definitions of meaningful treatment outcomes (Nweke & Eze 2019). The South African environment presents increased challenges for those with communication difficulties attempting to engage in successful social participation because of decreased awareness of communication disorders such as aphasia, alongside the lack of appropriate resources. These difficulties often result in misconceptions of an individual’s abilities, leading to reduced accessibility to their environments, including return to work (RTW), visiting shopping centres or making use of public transport (Barratt, Khoza-Shangase & Msimang 2012; Green, Mophosho & Khoza-Shangase 2015).

People with aphasia’s social participation may be further influenced by personal factors such as their age and role within society, as working-age adults (18–65 years of age) are often involved in a broader spectrum of activities compared to geriatric adults (Pike et al. 2017). It is estimated that up to 50% of strokes occur in adults aged 20 to 50 years (Krishnamurthi et al. 2015). Working-age adults face increased challenges compared to older PWA, as they are likely to live longer with the effects of aphasia, resulting in greater expectations of financial and social independence (Ntsiea, Van Aswegen & Olorunju 2013). This challenging phase of life requires PWA to re-engage in high-demand situations such as work, social situations, parental and spousal roles; however, they are often unable to fully reintegrate their premorbid roles, resulting in social impairments and social isolation (Alaszewski & Wilkinson 2015; Törnbom, Lundälv & Sunnerhagen 2019). Despite the high-demands faced by working-age adults with aphasia, there is limited evidence available regarding their experience of aphasia and their ability to re-engage in everyday life (Pike et al. 2017). One study indicates that working-age adults with aphasia express a desire to re-integrate within the community, employment, education, domestic life, social and leisure activities (Pike et al. 2017). Younger PWA are described to be intrinsically hopeful, resulting in increased motivation to reintegrate into everyday life, which may be because of the need to plan for their future and their demanding roles (Alaszewski & Wilkinson 2015). Contrastingly, the perspectives of social participation in older adults with chronic communication difficulties often demonstrate a reluctance to participate in activities because of feelings of demotivation and complacency as they have become accustomed to this new way of life (Alaszewski & Wilkinson 2015; Törnbom et al. 2019). An individual’s age, amount of time post-incident and stage of life play a key role in shaping social interests influencing perspectives of social participation (Ellis et al. 2019). These factors warrant the investigation of perspectives of working-age adults within the first 2 years post-incident.

It is evident that working-age adults present with contrasting experiences of social participation warrant individualised intervention. The need for person-centred care is highlighted, where SLTs are encouraged to facilitate functional treatment outcomes, resulting in increased quality of life (QoL) and valued social outcomes (Goodwin 2016). Literature shows that PWA are more likely to experience successful engagement in activities when rehabilitation targets realistic and personal goals (Haley et al. 2019; Törnbom et al. 2019). The ICF (WHO 2001) framework encourages SLTs to target various areas of PWAs’ everyday lives, thereby equipping them with the necessary and appropriate communication skills to participate in different social environments allowing meaningful outcomes (Elman 2016). There is a need for SLTs to implement holistic intervention based on PWAs’ personal perspectives, including their core values and interests, whilst considering the needs of the environmental context (Haley et al. 2019). Research demonstrates the need for continued attempts to understand the multiple factors involved in PWA’s experience of aphasia in LMIC, with the aim of improving guidance for healthcare professionals targeting meaningful and successful reintegration into everyday life (Legg & Penn 2013, 2014; Pike et al. 2017). There is great benefit in the adoption of culturally attuned frameworks of intervention, as it reinforces the principles of person-centred care and acknowledges personal factors alongside contextual environments (Penn & Armstrong 2017).

Person-centred care goes beyond focusing on the individual as it considers the role of PWA’s environment, using principles of community-based interventions, increasing community awareness of aphasia, allowing improved accessibility and empowering of PWA within their communities (Penn & Armstrong 2017). Speech-language therapists are in the ideal position to consider PWA’s communication abilities and needs allowing them to successfully participate in various social environments. Speech-language therapists are encouraged to obtain and target social participation through the implementation of dynamic dialogue between SLT and client, allowing SLTs to acknowledge PWA’s perspectives and target communication skills needed for their preferred social contexts. The aim of this study was to obtain working-aged PWA’s perspectives of social participation within the first 2 years post-incident in terms of what social activities, relationships and support PWA regard as meaningful, specific to their context.

## Research method and design

A phenomenological study was conducted using once-off semi-structured interviews with PWA. This approach recognises the complexity of the human experience, which is grounded in the world and experienced intersubjectively (Mason 2002; Neubauer, Witkop & Varpio 2019). This interpretive phenomenological approach (Tuffour 2017) allowed detailed examination and analysis of the lived and contextual experience of PWA gained through participant’s personal experiences and personal perception of social participation. This design allowed researchers to recognise, analyse and understand multiple facets that may contribute to a phenomenon rather than simplifying the problem. Facets such as context-specific barriers and facilitators present in PWA’s environment were considered (Leedy & Ormrod 2014) including their communication difficulties and the effects on social participation. A qualitative design was the most appropriate way to acknowledge and analyse the synergistic factors that may influence a complex phenomenon such as social participation (Törnbom et al. 2019).

### Participants

Ten adults with mild to moderate aphasia were recruited, using purposive sampling, as participants had to meet a specified inclusion criteria (Leedy & Omrod 2014). Participants were required to have a confirmed diagnosis of mild-moderate aphasia based on the results of the Western Aphasia Battery-Revised (WAB-R) (Kertesz 2009), as seen in Table 1. Participants had to be aged between 20 and 65 years old, with no other co-morbid disorders that may influence social participation, such as dementia or moderate-severe apraxia, as confirmed by their respective SLTs’ assessment results. The absence of hearing and visual impairments were ensured using the HearScreen™ (Swanepoel 2016) and Peek acuity (Bastawrous et al. 2015) smartphone applications. Table 1 reveals participants’ linguistic diversity demonstrating that although all participants spoke more than one language, English proficiency and understanding was ensured during interviews; screening tools and the interview schedule were compiled in English, as this is the lingua franca in South Africa (Khokhlova 2015). Participants had to be within the first 2 years post-incident to avoid possible compounded feelings of demotivation to participate in social activities, as often seen in individuals with chronic aphasia (Törnbom et al. 2019). This criterion was confirmed by participants’ respective SLTs’ case history and assessment results.

**TABLE 1:** Participant characteristics (*n* = 10).

Participant	Age	Months post-incident	Aphasia quotient	Severity	Cause	Gender	Languages spoken	Race	Profession pre-incident	RTW post-incident
P1	45	12	81,6	Mild	Stroke	Female	Afrikaans and English	White	Archaeologist	No
P2	57	22	86,6	Mild	Stroke	Female	Afrikaans and English	White	Executive manager	No
P3	55	24	59,3	Moderate	TBI and Stroke	Male	English and Afrikaans	White	Electrician	No
P4	58	13	55,65	Moderate	Stroke	Female	English	White	Human resources	No
P5	58	21	69	Moderate	Craniotomy	Female	English	White	School canteen manager	No
P6	40	8	78,5	Mild	Stroke	Male	Tswana and English	Black	Business development manager	Yes
P7	46	12	86,2	Mild	Stroke	Male	English and Portuguese	White - Portuguese	Geographic information specialist	Yes
P8	43	3	75	Mild	Stroke	Male	English and Afrikaans	Coloured	Entrepreneur	No
P9	39	11	78,1	Mild	Stroke	Male	Zulu, English and Tswana	Black	Firefighter	Yes
P10	42	6	86,2	Mild	Stroke	Male	English and Portuguese	White - Portuguese	Hospitality manager	No

RTW, return to work; TBI, traumatic brain injury.

### Data collection

Five SLTs working at private rehabilitation facilities were contacted in Gauteng, South Africa to recruit participants from their caseloads. Prospective participants were contacted by the first researcher and informed consent was obtained using principles from supported conversation techniques for adults with aphasia (SCA) (Kagan, Shumway & Podolsky 2010). Supports aided the PWA’s understanding of the study aim, as well as their proposed role in the study.

Once the screening of participants determined candidacy, a once-off semi-structured interview was conducted by the first researcher, a qualified SLT. The semi-structured interview enabled the researcher to follow the participant’s lead and explore the possible factors that may influence participant’s perspectives of a phenomenon, using open-ended questions, allowing the exploration of their thoughts, feelings and beliefs (DeJonckheere & Vaughn 2019; Nelson 2013). The interview consisted of initial closed-ended questions used to orientate the participants to the topics discussed in the open-ended questions, allowing consolidation and clarification of their information (Nelson 2013). The interview was compiled using existing literature on social participation in aphasia rehabilitation (Dalemans et al. 2008, 2010a, 2010b; Garcia & Connor 2011; Laliberté et al. 2016; Le Dorze et al. 2014; Wallace et al. 2017). Additionally, the Living with Aphasia: Framework for Outcome Measurement (A-FROM) (Kagan et al. 2008) was used to guide interview areas and corresponding questions. The A-FROM is an aphasia specific adaptation of the ICF that encourages SLTs to consider the impact of aphasia at a variety of levels, such as participants’ communication environment, personal attitudes and level of participation in activities. The semi-structured interview comprised of six areas including personal information, pre- and post-incident environments, feelings and attitudes, communication and language, and participation in life situations with family, friends and the community (Simmons-Mackie et al. 2014).

Participant’s interviews lasted approximately 1 h and were recorded using a Samsung A7 smartphone. Principles of SCA conversation for adults with aphasia techniques, pictorial aids and written supports were used in conjunction with the interview questions to augment participants’ ability to contribute and provide their perspectives (Murphy & Boa 2012). Principles of the SCA supported participants’ expressions of their perspectives using written aids and pictorial aids, whilst speaking at a slow pace (Kagan et al. 2010). Keywords regarding the subject of interview questions were written down on blank paper as a reference for participants. Pictorial aids facilitated sharing of opinions on specific topics by scaling participants’ feelings and satisfaction (i.e. happy, unsure and unhappy) and elaborating on these feelings (Murphy & Boa 2012). Participants could describe their level of satisfaction within the different areas of social participation by placing social participation activity pictures in a column associated with their level of satisfaction (happy, unsure or unhappy). This facilitated participants’ sharing of opinions and feelings on specific topics, whereby the researcher was able to prompt participants to further elaborate.

### Data analysis

Data were analysed using thematic analysis: a useful method for examining the perspectives of participants, highlighting similarities and differences, and possibly generating unanticipated insights (Braun & Clarke 2013; Leedy & Omrod 2014). Qualitative data analysis (QDA) Miner-Lite software (Provalis Research 2015) was used to code the data, which provided a dependable and effective measure for coding and categorising the dynamic factors involved in PWA’s perspectives of social participation.

Interviews were transcribed verbatim by the first researcher. Thematic analysis followed steps recommended by Braun and Clarke (2013). The first phase involved the first researcher reading the transcripts multiple times to identify common perspectives across the six areas evaluated in the semi-structured interview, such as the everyday routines, personal attitudes and beliefs, as well as relationships with family, friends and community (Laliberté et al. 2016). The second phase made use of QDA Miner-Lite software (Provalis Research 2015) to code specific extracts that formed the foundation of participants’ common perspectives. Common or like-minded perspectives were then highlighted and the respective extracts were coded under descriptors such as ‘desire to RTW’ or ‘supportive nature of significant other’. Phase three consisted of categorising the codes according to their overarching themes which described the sample’s perspectives of social participation, as demonstrated in Table 2.

**TABLE 2:** Example of coding tree used during data analysis.

Theme	Code	Example of data extract
Return to work	Work as social participation	‘I would love to work again, I would I really would because it’s it’s it’s good for you. It’s good to talk to people and to work and busy’ (P4)
	Desire to RTW	‘I feel I need to go back to work … to run my life you know?’ (P8)
	Job satisfaction	‘I was working most of the time but believe it or not, I’m one of those people who enjoys work’ (P10)
	Received support to RTW	‘I’m not yet full shift. I’m working on Monday to Friday and then on weekends – that’s when I’m off. Slowly, now they keep on engaging me to do even more work’ (P9)

RTW, return to work.

### Reliability, validity and trustworthiness

The value and rigor of this research study was accounted for by establishing trustworthiness, in which the researcher considered the credibility, dependability and confirmability of the results (Amankwaa 2016).

Qualitative data analysis Miner-Lite’s coding and categorisation features were used to verify connections between participant’s statements and overarching themes ensuring credibility (Amankwaa 2016; Provalis Research 2015). A peer-review analysis of the semi-structured interview schedule was conducted by an experienced external reviewer, ensuring confirmability and content validity, whilst reasserting the interview questions, responded to the study’s aim and was not hampered by researcher bias (Pyett 2003). The supplemental use of SCA techniques, pictorial and written aids acted as support for participants revealing their competence and increasing the confirmability and trustworthiness of participants’ responses (Kagan et al. 2010). Dependability of results were ensured during an extensive review process between the first researcher (N.M.S.) and the last researcher (B.S.P), where a consensus on themes was reached using peer debriefing, reflexive thoughts and revisiting the specific extracts from the raw data (Braun & Clarke 2013; Lincoln & Guba 1985). This ensured that the interpretation and development of themes were reflective of participants’ personal perspectives and related to the aim of the study.

### Ethical consideration

Institutional ethical clearance was granted by the Research Ethics Committee of the Faculty of Humanities, University of Pretoria (HUM043/05/19). Permission letters were sent to several SLTs working in the Gauteng area to recruit participants from private practices.

The study aim and procedures were explained in a manner prospective participants could understand, using principles of SCA (Kagan et al. 2010). Privacy and confidentiality were maintained throughout the research process. An alphanumeric code was assigned to each participant ensuring that no individual’s responses could be identified by anyone other than the researchers involved (Nelson 2013).

## Results

Seven main themes were identified according to the aim of the current study. Themes included participant’s personal attitudes towards social participation, the types of social activities and relationships participants (P) regarded as meaningful, as well as the importance of a supportive environment.

### Main themes

#### Preferred communication partners

Eight of the 10 participants (80%) preferred spending time with family members or close friends, as opposed to meeting new people or attending larger social events. Some participants stated that they were not inclined to, or want to meet new people because of their existing friendships being satisfactory, whilst others expressed feelings of anxiety regarding possible communication breakdowns. It appears that the level of familiarity between the participant and communication partner, the duration of their relationship and the supportive nature of the communication partner influenced participants’ willingness to socialise post-incident, as seen in the statements below:

‘I must say, they understand. X (friend) (for) instance is one of my best friends and she, you know, picks it up and it’s amazing how they pick up. They understand what I wanna say when I can’t say it and they say “is it that or that?” They understand.’ (P1)

‘You don’t talk too much with new people…because they don’t know me. (But) People if I can, if I can do certain things…those that know me…if I say something, they will offer you help … whereas strange people won’t *sommer* [Afrikaans word – local language, meaning “simply”], you don’t play with - you don’t p-talk to them, it’s different.’ (P4)

‘Well they (wife and best friend) um, I mean the thing is they say, well, they see the improvements and they…that’s what they focus on and they don’t say “oh no, you not doing great.”’ (P7)

The remaining two participants (20%) were open to attending social events and engaging with new individuals in the future, when they are able to independently communicate. In the meantime, these two participants were willing to meet new people provided their significant others were present to facilitate and support conversations.

#### Return to work

Seven participants (70%) expressed a desire to RTW yet only three participants (30%) were able to do so. The reduced rate of RTW highlights enforced early retirement because of aphasia.

Participants expressed feelings of previous job satisfaction and described themselves as ‘busy’ people pre-incident whereby they missed having stimulating tasks, or co-workers to interact with, as demonstrated by P4’s statement:

‘I would love to work again, I would, I really would because it’s it’s it’s good for you.’ (P4)

Most participants expressed a willingness to RTW because of a desire to keep busy and interact with others in a work setting, as opposed to staying home, as seen in the statement by P7:

‘If it wasn’t for work, I’d be at home and what would I do at home? Who’s …who is here? There’s no one …’ (P7)

Participant 7 went on to explain that simple conversations with colleagues were missed, as seen in the following quote:

‘Even the colleagues, I don’t I don’t so-socialise with them a lot, but they-they do say hi and then I say hi back.’ (P7)

The participants who were able to RTW (30%) were men diagnosed with mild aphasia, with a mean age of 42 years. Certain pertinent factors appeared to contribute to reintegrating into the work environment. In the cases of P7 and P9, their places of work implemented adaptations to their roles through the assignment of less demanding tasks, reduced caseloads and flexible working hours. For P6, he was able to RTW as he received direct support from his significant other in managing his business. Those that did not express desire to RTW (*n* = 30%) stated that work was not a priority at that moment, as they preferred to prioritise meaningful tasks such as spending quality time with family, carrying out previous hobbies or interests or they simply felt they ‘had worked enough’ and may have been closer to the age of retirement.

#### Faith-related activities

Seven participants (70%) explained that faith-related activities, such as attending church or bible study groups, were important parts of their weekly social participation routine. Faith-related activities were found to be the primary social activity amongst participants, which included social participation with members other than the participants’ immediate family or close friends. Participants looked forward to attending faith-related social gatherings as they felt comfortable, supported and safe in this environment. P3 explained that he felt safe in church but did not always feel comfortable in other settings, and stated: ‘I talk to everybody in the church, but outside you can’t do that, people don’t do that, but in church, you talk to me and I talk to everyone’.

Participants also explained a heightened sense of purpose to help others. This is illustrated by the following statement by P1: ‘In a sense I’m glad that I had the stroke, because without it wouldn’t have *saved* me [Christian term used to explain renewed belief in God]. There is obviously a meaning behind it and God said he has got a plan for me. I talk to people who had a stroke and I tell them “it’s going to get better, I’ve been through it” …’.

The remaining three participants (30%) did not consider faith-related activities an important area of social participation and did not engage in faith-related activities often; rather their free time was spent with family and/or close friends.

#### Value of rehabilitation services

Participants received several rehabilitation services, including speech-language therapy (ST), physiotherapy and occupational therapy. Nine participants (90%) mentioned that therapy was a part of their weekly routines, of which 20% stated physiotherapy exercises were a part of their weekly routines and 70% reported that ST exercises and home programmes became part of their daily and/or weekly social routine. It appears that SLTs may play a role in providing feelings of optimism and motivation for improvement in the future, as highlighted by the following:

‘I wake up in the morning, I have breakfast and then I’ll go with the things (gestures to ST home programme), I read the books, it’s just therapy at the moment (gestures to ST home programme again).’ (P10)

‘Ya *eish* [colloquial exclamation – expressing emotion], I changed, even my communication skills, I hope they will help me here… (gestures to ST private practice).’ (P6)

Participants frequently reported they performed their home programmes with the assistance of a caregiver or significant other, indicating a case of social participation. Additionally, it appears that participants’ ST sessions and the home programmes became an opportunity for social participation, as seen by the statements below:

‘Most of the time, especially if um Y (SLT) gave me some homework…then we (wife, child and participant) sit and discuss about it and then they hear my views and what do I have to say.’ (P9)

‘Oh I love it (ST)! Z (SLT) we have such nice fun… I tell you but we’ve had such giggles!’ (P4)

These findings highlight the possible role of the SLTs in PWA’s social network, as well as the influence of rehabilitative services in shaping participants’ perspectives of social participation, fostering hope and motivation, whilst providing facilitating social participation in weekly routines.

#### Returning to previous interests

Participants’ pre-incident interests guided their preference for the type of activities they engaged in post-incident. Participants were questioned regarding their leisure time and their willingness to engage in new or unfamiliar social activities. All participants either continued participating in previous activities, such as spending time with old friends or family, gardening, cooking or other work, or they expressed a desire to re-engage in previous social activities as opposed to engaging in new or unfamiliar activities. P9 specifically stated that he had not started engaging in new activities as he felt he was ‘not ready yet’ and preferred to direct his focus on his preferred activities he engaged in prior to the incident, such as work and spending time with family, before he could start new activities. This was reiterated in the following statement by P9: ‘First thing is, I must get fixed you know then I will see…then I can decide what I want to do’.

Findings demonstrated participants’ preference for the quality of activities as opposed to the quantity thereof, in which participants explained they would prefer to see improvement in their previous interests before commencing new activities.

#### Presence of support from others

Most participants (80%) were more inclined to attend social events and visit public areas if they were accompanied by a caregiver or significant other. For example, P9 stated he would rather stay home and would only go to new places if accompanied by his spouse, as he described it to be ‘pretty necessary’ for his wife to help him communicate. Notably, P9 was diagnosed with mild aphasia and was able to RTW; however, he still did not feel comfortable participating independently in new environments. This feeling was reiterated by 50% of participants, who reported a desire for support because of possible communication breakdowns with unfamiliar communication partners. Some participants (30%) were also reliant on physical support from their significant others, such as driving to public places.

#### Positive attitudes and feelings

Nine participants (90%) expressed feelings of hope for improved communication and participation. Participants described their eagerness to function as close as possible to their pre-morbid lifestyle and to participate more frequently in social activities once they experienced significant improvement. Specifically, participants did not appear to view their future negatively, but rather looked forward to reaching a level of functioning where they felt comfortable to participate with family, friends and within the community, as demonstrated by the following statements:

‘I still want to meet new people, because I practice every day.’ (P6)

‘It (aphasia) make me so sad but it’s one or the other, at the end of the day you need uh, umm you need to try-try and give your all, give it your all.’ (P9)

A positive attitude towards the future was noticed throughout the interviews, where participants appeared to have a heightened sense of purpose and hope. Participants reached a level of understanding of their current circumstances, in which they were satisfied with their present level of participation, whilst still being determined to achieve functional improvements.

## Discussion

The results of this study indicated that social participation as viewed by participants of this study was determined by the perceived value of activities and their willingness to participate. Results further demonstrated that perspectives of meaningful social participation are shaped by personal factors, such as PWA’s feelings and attitudes towards a social situation, communicative skills and confidence. Environmental factors were found to play a role in forming perspectives, particularly the supportive nature of communication partners and the latter’s communication skills, alongside attitudes of community members towards aphasia as well as accessibility to different environments.

Participants described an overall attitude of positivity and motivation towards the future and engagement in social activities, as they expressed a willingness to interact more with family, friends and within their community, once they feel confident. This motivation may be attributed to their faith, as indicated by 70% of participants. Research demonstrates that faith may contribute to improved recovery because of its provision of a sense of identity and increased feelings of hope, strength and support (Laures-Gore et al. 2018; Masuku & Khoza-Shangase 2018). The increased levels of hope may also be explained by the younger age of the sample, as it is evident that the experience of aphasia differs between working-age adults with aphasia and older individuals (Alaszewski & Wilkinson 2015). The impact of the onset of aphasia and the change experienced is arguably more abrupt in working-age adults because of their highly demanding stage of life. This may explain the increased motivation to return to a pre-morbid level of functioning and independence sooner, rather than later (Alaszewski & Wilkinson 2015). Additionally, enthusiasm may be attributed to the minimal number of years post-stroke, as PWA 7 to 8 years post-incident reported contrasting feelings, where they were satisfied with the changes in their everyday routine and more accepting of their level of functioning (Törnbom et al. 2019). The definition of living successfully with aphasia must undergo a paradigm shift from managing the effects of aphasia towards participation in everyday life to the best extent possible (Manning et al. 2017). People with aphasia who adopted positive attitudes of hopefulness and motivation are more likely to experience success when attempting to re-engage in their hobbies and social activities (Dalemans et al. 2010b; Woodman et al. 2014). It is the role of health care professionals, such as SLTs to acknowledge the importance of these attitudes and behaviours in promoting meaningful rehabilitation outcomes (Woodman et al. 2014) through facilitating opportunities for social participation and improved communication abilities.

### Preference for communication partners

A decline in participants’ social networks post-incident was reported upon the diagnosis of aphasia, resulting in a preference for specific communication partners. Participants were more inclined to engage with family and close friends with whom they experienced greater conversational ease. The characteristics of communication partners significantly impact PWA’s willingness to socialise, whereby increased levels of familiarity and duration of a relationship with a significant other result in PWA being willing to participate socially (Dalemans et al. 2010a). People with aphasia demonstrate a preference for meaningful social situations specifically time with family and close friends, where they feel understood, accepted and supported (Manning et al. 2017), as demonstrated by the findings of the current study. It has been found that PWA’s communication confidence and their ability to pre-empt communication breakdowns impact the likelihood of their participation in social situations. Communication partners can support PWA’s confidence and help manage communication breakdowns, increasing PWA’s motivation to participate, in turn facilitating social participation (Chiou & Yu 2018).

Speech-language therapists must facilitate not only the PWA’s communication abilities but also the role of significant others in attaining successful social participation. Acknowledgement of PWA’s context and individualised goals are reflected in the principles of person-centred care, allowing for a greater understanding of personal and environmental challenges (Goodwin 2016). Speech-language therapists are encouraged to target not only the communication impairment but also the PWA’s feelings of confidence and motivation to participate, as well as the conversational skills of the significant others through the use of conversational partner training (Chiou & Yu 2018; Simmons-Mackie, Raymer & Cherney 2016).

### Return to previous interests

Personal factors continued to shape participants’ perspectives of social participation, including their preference for pre-incident activities. Participants expressed a desire to re-engage in previous interests, which was reiterated in their willingness to continue interacting with family and close friends, return to previous leisure activities and engage faith-related activities rather than pursuing novel experiences. People with aphasia have demonstrated a desire to improve their ability to converse within group settings and discussions, as well as to increase their social networks (Wallace et al. 2017). However, participants in the current study expressed reluctance for increased involvement in social situations, with a preference for improvement in the activities they engaged in pre-incident. The value of quality interactions over quantity is well documented in literature, reinforcing the importance of PWA’s feelings and attitudes in shaping their perspectives of social participation (Dalemans et al. 2010a; Howe 2017; Törnbom et al. 2019). Speech-language therapists need to acknowledge PWA’s pre-morbid preferences, together with personal and environmental factors that influence their decision to engage in certain social activities as opposed to others, thus, allowing the implementation of person-centred care and empowering PWA with context-specific communication skills (Dalemans et al. 2010a).

### Return to work

Participants expressed a desire to RTW as it provided them with a sense of purpose within their weekly routines, alongside opportunities for social participation with individuals who were not family and friends. The work environment provides PWA with opportunities to increase their communicative confidence, overall QoL and maintain their social identity (Callander & Schofield 2013) as demonstrated in participants’ explanation of the importance of their work environment in maintaining social networks and sense of purpose. Social support has been acknowledged as a crucial determinant in an individual’s ability to RTW (Törnbom et al. 2019). Results of the present study reiterated that the presence of support from the workplace allowed participants to RTW, whereby employers adjusted participants’ roles and working environments to suit their post-morbid abilities. The majority of PWA who RTW did so within the first 18 months post-incident (Ntsiea et al. 2013) suggesting that the remaining 70% of participants may still RTW. However, increased amount of time post-incident, increased age and the diagnosis of moderate aphasia (Aarnio et al. 2018) are factors associated with the reduced likelihood of PWA returning to work. This may explain why some of the older participants in the sample have not returned to work or did not want to RTW. In South Africa, there is scant awareness of aphasia and limited accessibility within community environments (Green et al. 2015), providing another possible explanation for the reduced rate RTW amongst participants. This finding highlights the importance of advocacy and awareness within the South African context, in order to improve PWA’s ability to RTW, in turn facilitating social participation and QoL. Communication partner training and increased awareness of aphasia are instrumental in improving PWA’s chances of successfully participating within the community, including returning to work (Simmons-Mackie et al. 2016; Törnbom et al. 2019). Results emphasise the value of work as an area of social participation, emphasising the need for SLT’s to target PWA’s vocational communication abilities alongside their social communication skills needed in their work environment. Speech-language therapists are encouraged to advocate the importance of returning to work for PWA, because of its correlation with improved QoL and social participation (Edwards et al. 2018; Howe 2017).

### Faith-related activities

Feelings of comfort and support in environments were found to be a strong determinant of participants’ choice of social engagements and perspectives of meaningful social participation. Participants demonstrated minimal involvement within the community other than engagement in faith-related activities, such as church and bible study. One study found that the only stroke-related factor that continues to contribute to a decline in PWA’s social networks 6 months post-incident is aphasia severity (Northcott, Marshall & Hilari 2016). However, results of the current study demonstrated the decline is not only because of the diagnosis of aphasia but also participants’ feelings and attitudes. Faith-related activities provide individuals with a sense of purpose, feelings of inclusivity, and in turn provide social support (Masuku & Khoza-Shangase 2018), thus possibly explaining participants’ choice of engaging with these particular situations, as opposed to others within the community. The relationship between spirituality, social participation and aphasia has not been extensively researched. However, faith-related activities have been recognised as opportunities for PWA to communicate with individuals outside their immediate environment (Laures-Gore et al. 2018). This was supported by participants’ descriptions of faith-related activities as an area of social participation, whilst highlighting the importance of feeling comfortable and supported within social situations.

The value of faith-related activities in South Africa is evident within this sample, as participants explained a heightened sense of purpose to help others, whilst strengthening their faith post-incident. This sense of purpose for PWA has not specifically been linked to spirituality or faith in literature, but rather to feelings of gratitude for surviving and a willingness for engagement in meaningful activities (Törnbom et al. 2019). This difference may be attributed to the contrasting perceptions and understanding of illness and incidents such as stroke, across geographical regions and culture. Literatures originating from HICs explain that the illness is predominately attributed to medical reasons. However, in LMICs, such as South Africa, faith plays an undeniable role in individuals’ definition of illness (Nweke & Eze 2019). Additionally, PWA describe feelings of confidence and support within their faith community, in which they feel comfortable socialising with others (Masuku & Khoza-Shangase 2018) as demonstrated in this study. This finding emphasises the influence of cultural and contextual factors in shaping participants’ perspectives of social participation.

Hope is fostered in the presence of social support and within interpersonal relationships (Bright, McCann & Kayes 2019), highlighting the influence that significant others and faith have in facilitating increased feelings of positivity in participants, resulting in improved social participation and QoL. Speech-language therapists must acknowledge the possible role of faith in facilitating motivation and hope, whilst attempting to empower participants’ ability to successfully communicate in social situations they consider meaningful. Participants’ perspectives from the current study, alongside previous literature, stress the importance of considering faith-related activities when setting goals and attempting to implement holistic intervention (Masuku & Khoza-Shangase 2018).

### Value of rehabilitation services

Rehabilitation services, including ST and physiotherapy, were identified as valued forms of social participation in participants’ weekly routines. Literature has identified a lack in the ability of rehabilitation services to support and target younger PWA’s parental roles (Manning et al. 2017). Contrastingly, the results of this study indicated home programmes were often completed with the help of the PWA’s child and significant other, in turn providing opportunities for engagement within the home environment. The results shed light on the possible positive influence of rehabilitation services, specifically speech-language therapy, in social participation. Participants described positive relationships with their SLTs, where they appreciated opportunities for conversation practice within therapy sessions. Positive therapeutic relationships with SLTs are recognised across literature, as PWA often feel understood, empowered and supported by their SLTs (Lawton et al. 2018; Manning et al. 2017; Northcott et al. 2018). Social connections and relationships often form between client and SLT, because of the interrelated nature of targeting improved functional communication, whilst considering the clients’ emotional well-being and implementing relational practices, such as getting to know one another and carrying out ‘small talk’ (Lawton et al. 2018; Northcott et al. 2018). The findings from this study demonstrate the role of SLTs in facilitating social participation, particularly in the first few years of recovery, as well as their personal role as a member of their client’s social network.

The benefits of positive therapeutic relationships were supported in this study, where participants explained increased motivation and psycho-social well-being (Lawton et al. 2018). However, South Africa is made up of a linguistically diverse population resulting in a shortage of SLTs who are proficient in clients’ first language, as the majority of SLTs only speak English and Afrikaans (Barratt et al. 2012). The language barrier and the shortage of SLTs highlight the possible challenge in creating meaningful relationships between SLTs and their clients, as well as the difficulty in achieving effective person-centred care in South Africa.

Community-based service delivery, specifically group therapy, is encouraged in South Africa because of its provision of creative and culturally-rich stimulation that acknowledges context, whilst targeting a number of individuals in an authentic social manner; thus addressing the lack of appropriate ST services (Penn 2014). Conversational aphasia groups have demonstrated significant improvement in PWA’s communication abilities, whilst providing an opportunity for authentic social participation with others of similar circumstance (Penn & Armstrong 2017). Implementation of group therapy may significantly improve PWA’s social participation, through the development of new social networks and increased social support whilst targeting their communication impairments (Howe 2017; Penn & Armstrong 2017).

## Limitations of current study

The first researcher is an expatriate who is proficient in English, with some understanding of Afrikaans, thus making it difficult to interview a complete profile of South Africa’s diverse population without including a translator. It is limiting that the current sample does not represent the full diversity of the South African context. However, small sample sizes are prevalent in qualitative research as smaller sample sizes often represent a large quantum of in-depth information collected (Nelson 2013). Nevertheless, this study may contribute to the expansion of evidence regarding social participation in LMIC (Pike et al. 2017). The researcher met with the participants only once and had limited verbal interaction with them prior to the semi-structured interviews, possibly leading to participants’ sharing less. Longitudinal and multiple investigations of PWA’s perspectives may allow for greater understanding of the barriers and facilitators of social participation experienced, over an increased period. Study findings shed light on what PWA may perceive as successful social participation, as well as the possible areas of social participation specific to this context, such as relationships with family, friends, SLTs and members of their faith community. Due to the heterogeneity of PWA in South Africa, similar investigations must continue and be broadened to include larger and more diverse samples, to inform person-centred care and optimise functional outcomes post-incident.

## Conclusion

It is clear that feeling included, comfortable and supported can shield an individual from experiencing social isolation (Elloker & Rhoda 2018), in turn allowing PWA to experience improved confidence in communication and social participation. SLTs play a unique role in the implementation of community-led intervention in South Africa, which may significantly improve PWA’s social participation. The involvement of significant others and community members, such as family and faith leaders in intervention is encouraged alongside the introduction of advocacy and awareness programs (Watermeyer 2019) to improve feelings of comfort and conversational confidence. It is the role of SLTs to advocate for increased communication partner and vocational training, allowing improved accessibility for PWA and facilitating social participation. Speech-language therapists must support communication abilities needed for PWA to engage in social activities of interest and value, leading to meaningful social participation (Woodman et al. 2014). The findings of this research demonstrate the importance of optimising PWA’s increased feelings of motivation and hope through the stages of recovery, and the role of person-centred care in achieving successful social participation for PWA.
